# Supplementary material for: Melatonin Supplementation Alters Maternal and Fetal Amino Acid Concentrations and Placental Nutrient Transporters in a Nutrient Restriction Bovine Model
Source: Metabolites. 2022 Dec 2;12(12):1208. doi: 10.3390/metabo12121208 (PMC9782144; doi:10.3390/metabo12121208)
Supplement: Supplementary file 1 [file metabolites-12-01208-s001.zip › metabolites-2045871-supplementary.pdf]

**Supplemental Table S1.** Individual amino acids summed for analysis by transport system and essential groups.

| System | Amino Acids Included |
|---|---|
| **System A** | Alanine, serine, glutamine |
| **System Br** | Valine, leucine, isoleucine |
| **System Bo** | Phenylalanine, tyrosine, tryptophan, valine, leucine, isoleucine |
| **System N** | Histidine, aspartic acid, glutamine |
| **Cationic** | Lysine, ornithine |
| **Anion** | Aspartic acid, glutamic acid |
| **Essential** | Valine, leucine, isoleucine, threonine, methionine, phenylalanine, lysine, histidine |

**Supplemental Table S2.** Individual amino acids in maternal plasma in the fall replicate.

| Amino Acid (µmol/L) | ADQ-CON | RES-CON | ADQ-MEL | RES-MEL | *P* value NUT | *P* value TRT | *P* value NUT*TRT |
|---|---|---|---|---|---|---|---|
| Alanine | 186.58 ± 15.10$^{b}$ | 345.87 ± 63.03$^{a}$ | 235.96 ± 42.49$^{a}$ | 188.55 ± 11.22$^{a}$ | - | - | **0.05** |
| Sarcosine | 6.97 ± 0.28$^{c}$ | 8.82 ± 0.62$^{a}$ | 7.73 ± 0.40$^{abc}$ | 7.30 ± 0.26$^{b}$ | - | - | **0.01** |
| Glycine | 328.15 ± 41.74 | 324.81 ± 18.66 | 293.50 ± 13.98 | 315.74 ± 35.45 | 0.19 | 0.38 | 0.83 |
| α-Aminobutyric acid | 14.38 ± 1.54$^{b}$ | 25.04 ± 3.88$^{a}$ | 17.71 ± 1.56$^{ab}$ | 17.15 ± 2.14$^{ab}$ | - | - | **0.02** |
| Valine | 227.66 ± 12.59 | 201.01 ± 12.59 | 254.09 ± 12.59 | 238.16 ± 11.74 | 0.10 | **0.02** | 0.67 |
| β-Aminoisobutyric acid | 7.46 ± 0.18 | 9.81 ± 1.20 | 7.84 ± 0.43 | 7.36 ± 0.33 | 0.99 | 0.28 | 0.14 |
| Leucine | 123.56 ± 7.76 | 103.89 ± 7.76 | 125.95 ± 7.76 | 127.14 ± 7.24 | 0.24 | 0.11 | 0.18 |
| Isoleucine | 88.03 ± 7.54 | 68.52 ± 7.54 | 90.35 ± 7.54 | 89.50 ± 7.03 | 0.18 | 0.13 | 0.22 |
| Threonine | 44.12 ± 3.84 | 42.42 ± 3.79 | 54.96 ± 3.79 | 45.05 ± 3.59 | 0.14 | 0.09 | 0.28 |
| Serine | 43.57 ± 4.68 | 58.08 ± 4.62 | 51.60 ± 4.62 | 48.84 ± 4.38 | 0.21 | 0.90 | 0.07 |
| Proline | 60.81 ± 4.77 | 88.85 ± 9.44 | 70.96 ± 9.46 | 66.06 ± 5.30 | 0.11 | 0.75 | 0.06 |
| Asparagine | 20.59 ± 1.71 | 26.20 ± 1.74 | 23.89 ± 1.76 | 24.19 ± 1.59 | 0.09 | 0.71 | 0.14 |
| Thiaproline | 2.50 ± 0.42 | 2.11 ± 0.55 | 1.25 ± 0.59 | 1.87 ± 0.55 | 0.79 | 0.14 | 0.79 |
| Aspartic acid | 10.12 ± 0.22$^{b}$ | 19.54 ± 3.82$^{a}$ | 12.20 ± 1.05$^{a}$ | 10.68 ± 0.58$^{ab}$ | - | - | **<0.01** |
| Methionine | 18.23 ± 0.96 | 19.48 ± 0.97 | 21.47 ± 0.97 | 19.70 ± 0.89 | 0.78 | 0.08 | 0.13 |
| 4-Hydroxyproline | 21.01 ± 0.95 | 55.56 ± 13.23 | 29.90 ± 10.75 | 21.76 ± 1.14 | **0.04** | 0.36 | 0.63 |
| Glutamic acid | 66.60 ± 2.43$^{c}$ | 224.84 ± 59.45$^{a}$ | 95.48 ± 16.06$^{ab}$ | 79.33 ± 11.68$^{bc}$ | - | - | **<0.01** |
| Phenylalanine | 45.85 ± 1.78 | 60.94 ± 6.81 | 50.37 ± 3.83 | 48.87 ± 2.51 | 0.14 | 0.95 | 0.18 |
| α-Aminoadipic acid | 10.16 ± 0.28$^{b}$ | 12.31 ± 0.56$^{a}$ | 10.77 ± 0.54$^{ab}$ | 10.32 ± 1.99$^{b}$ | - | - | **0.03** |
| Glutamine | 280.27 ± 33.54$^{b}$ | 635.50 ± 110.09$^{b}$ | 398.92 ± 78.03$^{ab}$ | 278.39 ± 59.15$^{b}$ | - | - | **<0.01** |
| Ornithine | 48.63 ± 4.56 | 53.54 ± 4.56 | 58.42 ± 4.56 | 48.82 ± 4.26 | 0.60 | 0.57 | 0.12 |
| Glycine-proline | 0.03 ± 0.02 | 1.90 ± 0.73 | 0.84 ± 0.83 | 0.13 ± 0.09 | 0.17 | 0.22 | 0.09 |
| Lysine | 60.34 ± 5.75$^{b}$ | 75.75 ± 5.75$^{a}$ | 71.36 ± 5.75$^{ab}$ | 57.50 ± 5.36$^{b}$ | - | - | **0.02** |
| Histidine | 41.18 ± 5.58 | 48.50 ± 5.58 | 47.02 ± 5.58 | 36.37 ± 5.20 | 0.76 | 0.57 | 0.11 |
| Hydroxylysine | 8.84 ± 0.11$^{ab}$ | 9.29 ± 0.20$^{a}$ | 9.08 ± 0.16$^{ab}$ | 7.76 ± 1.13$^{b}$ | - | - | **0.02** |
| Tyrosine | 40.75 ± 3.98$^{b}$ | 52.05 ± 3.98$^{a}$ | 49.87 ± 3.98$^{ab}$ | 41.88 ± 3.71$^{ab}$ | - | - | **0.02** |
| Proline-hydroxyproline | 19.12 ± 0.38 | 36.92 ± 7.55 | 26.39 ± 7.58 | 19.75 ± 0.39 | **0.02** | 0.78 | 0.54 |
| Tryptophan | 33.56 ± 2.58 | 35.51 ± 2.58 | 35.30 ± 2.58 | 29.07 ± 2.41 | 0.41 | 0.37 | 0.12 |
| Cystathionine | 0.12 ± 0.12$^{ab}$ | 4.04 ± 2.02$^{a}$ | 0.92 ± 0.77$^{ab}$ | 0.00 ± 0.00$^{b}$ | - | - | **0.04** |
| Cystine | 16.24 ± 1.12$^{bc}$ | 19.29 ± 1.44$^{ab}$ | 19.03 ± 1.03$^{a}$ | 11.40 ± 2.52$^{c}$ | - | - | **<0.01** |

Data are presented as means ± standard error. Bolded *P* values are significant at $P \leq 0.05$. When there is a significant interaction, the *P* values for main effects of nutrition and treatment are not shown and replaced with a dash (-). Superscripts(a,b,c) denotes differences at $P \leq 0.05$. Concentrations are expressed in µmol/L. Experimental units: ADQ-CON, n = 6; RES-CON, n = 6; ADQ-MEL, n = 6; RES-MEL n = 7. Abbreviations: NUT, nutrition; TRT, treatment; NUT*TRT, nutrition by treatment interaction; ADQ-CON, adequately fed control; RES-CON, restricted fed control; ADQ-MEL, adequately fed melatonin supplemented; RES-MEL, restricted fed melatonin supplemented.

**Supplemental Table S3.** Individual amino acids in fetal plasma in the fall replicate.

| Amino Acid (µmol/L) | ADQ-CON | RES-CON | ADQ-MEL | RES-MEL | *P* value NUT | TRT | NUT*TRT |
|---|---|---|---|---|---|---|---|
| Alanine | 454.89 ± 40.12 | 453.04 ± 40.12 | 459.44 ± 40.12 | 432.80 ± 37.43 | 0.72 | 0.84 | 0.76 |
| Sarcosine | 9.91 ± 0.73 | 9.77 ± 0.54 | 10.13 ± 0.89 | 9.25 ± 0.44 | 0.53 | 0.56 | 0.93 |
| Glycine | 328.83 ± 18.73 | 300.82 ± 18.89 | 291.32 ± 19.72 | 325.88 ± 17.94 | 0.87 | 0.74 | 0.10 |
| α-Aminobutyric acid | 28.33 ± 3.70 | 32.68 ± 3.70 | 30.43 ± 3.70 | 28.39 ± 3.45 | 0.75 | 0.77 | 0.39 |
| Valine | 210.29 ± 18.80 | 211.51 ± 18.96 | 200.31 ± 19.79 | 218.02 ± 18.01 | 0.65 | 0.93 | 0.66 |
| β-Aminoisobutyric acid | 11.78 ± 0.87 | 10.38 ± 0.87 | 10.59 ± 0.87 | 11.63 ± 0.81 | 0.84 | 0.97 | 0.17 |
| Leucine | 90.21 ± 6.67 | 84.93 ± 6.85 | 86.34 ± 7.13 | 84.44 ± 6.31 | 0.62 | 0.75 | 0.80 |
| Isoleucine | 57.33 ± 4.76 | 57.41 ± 4.89 | 54.63 ± 5.08 | 53.75 ± 4.50 | 0.94 | 0.51 | 0.92 |
| Threonine | 49.72 ± 3.29 | 37.97 ± 3.78 | 51.83 ± 5.11 | 35.01 ± 5.64 | 0.18 | 0.71 | 0.20 |
| Serine | 75.12 ± 7.65 | 71.53 ± 8.26 | 77.74 ± 8.13 | 75.95 ± 7.20 | 0.75 | 0.65 | 0.91 |
| Proline | 103.89 ± 6.18 | 105.37 ± 6.35 | 101.53 ± 6.61 | 100.25 ± 5.85 | 0.99 | 0.56 | 0.82 |
| Asparagine | 24.50 ± 1.25 | 25.76 ± 1.25 | 24.27 ± 1.25 | 23.36 ± 1.17 | 0.89 | 0.30 | 0.39 |
| Thiaproline | 1.26 ± 0.59 | 2.08 ± 0.54 | 1.25 ± 0.59 | 1.82 ± 0.53 | 0.59 | 0.98 | 0.99 |
| Aspartic acid | 23.81 ± 1.79 | 18.51 ± 2.31 | 26.00 ± 3.01 | 24.56 ± 2.36 | 0.23 | 0.15 | 0.66 |
| Methionine | 20.52 ± 1.41 | 19.69 ± 1.42 | 23.14 ± 1.43 | 18.28 ± 1.32 | **0.05** | 0.67 | 0.17 |
| 4-Hydroxyproline | 77.53 ± 6.31 | 78.69 ± 6.31 | 83.48 ± 6.31 | 75.15 ± 5.88 | 0.57 | 0.85 | 0.45 |
| Glutamic acid | 306.37 ± 38.63 | 262.23 ± 39.69 | 362.37 ± 41.29 | 377.23 ± 36.54 | 0.72 | **0.04** | 0.45 |
| Phenylalanine | 81.70 ± 5.30 | 78.67 ± 5.72 | 70.03 ± 5.63 | 81.53 ± 4.99 | 0.47 | 0.41 | 0.19 |
| α-Aminoadipic acid | 13.31 ± 0.38 | 12.90 ± 0.43 | 13.26 ± 0.46 | 14.32 ± 1.30 | 0.44 | 0.85 | 0.36 |
| Glutamine | 696.38 ± 121.28 | 690.72 ± 121.28 | 808.77 ± 121.28 | 730.39 ± 113.17 | 0.73 | 0.53 | 0.76 |
| Ornithine | 55.61 ± 6.43 | 58.69 ± 6.54 | 69.40 ± 6.61 | 62.15 ± 5.97 | 0.74 | 0.19 | 0.44 |
| Glycine-proline | 3.85 ± 0.72 | 2.32 ± 0.74 | 3.70 ± 0.77 | 3.00 ± 0.68 | 0.16 | 0.72 | 0.57 |
| Lysine | 78.50 ± 7.52 | 73.75 ± 7.52 | 81.35 ± 7.52 | 67.21 ± 7.02 | 0.21 | 0.81 | 0.53 |
| Histidine | 49.63 ± 6.48 | 35.90 ± 6.48 | 50.42 ± 6.48 | 38.65 ± 6.05 | 0.06 | 0.78 | 0.88 |
| Hydroxylysine | 9.81 ± 0.22 | 9.75 ± 0.26 | 10.02 ± 0.24 | 9.94 ± 0.21 | 0.83 | 0.32 | 0.96 |
| Tyrosine | 63.30 ± 5.73 | 63.25 ± 6.18 | 60.70 ± 6.09 | 59.43 ± 5.39 | 0.92 | 0.58 | 0.92 |
| Proline-hydroxyproline | 55.84 ± 5.60 | 45.04 ± 5.60 | 58.51 ± 5.60 | 47.61 ± 5.23 | 0.06 | 0.64 | 0.99 |
| Tryptophan | 42.94 ± 3.94 | 38.64 ± 3.94 | 41.63 ± 3.94 | 32.47 ± 3.67 | 0.09 | 0.35 | 0.54 |
| Cystathionine | 6.81 ± 1.92 | 5.59 ± 1.92 | 8.58 ± 1.92 | 7.59 ± 1.79 | 0.56 | 0.33 | 0.95 |
| Cystine | 18.72 ± 0.48 | 17.92 ± 1.29 | 20.20 ± 1.22 | 15.78 ± 2.37 | 0.13 | 0.71 | 0.32 |

Data are presented as means ± standard error. Bolded *P* values are significant at $P \leq 0.05$. When there is a significant interaction, the *P* values for main effects of nutrition and treatment are not shown and replaced with a dash (-). Superscripts(a,b,c) denotes differences at $P \leq 0.05$. Concentrations are expressed in µmol/L. Experimental units: ADQ-CON, n = 6; RES-CON, n = 6; ADQ-MEL, n = 6; RES-MEL n = 7. Abbreviations: NUT, nutrition; TRT, treatment; NUT*TRT, nutrition by treatment interaction; ADQ-CON, adequately fed control; RES-CON, restricted fed control; ADQ-MEL, adequately fed melatonin supplemented; RES-MEL, restricted fed melatonin supplemented.

**Supplemental Table S4.** Individual amino acids in amnion in the fall replicate.

| Amino Acid (µmol/L) | ADQ-CON | RES-CON | ADQ-MEL | RES-MEL | *P* value NUT | TRT | NUT*TRT |
|---|---|---|---|---|---|---|---|
| Alanine | 61.35 ± 13.73[b] | 156.58 ± 27.80[a] | 209.48 ± 80.92[a] | 108.93 ± 50.15[ab] | - | - | **<0.01** |
| Sarcosine | 5.01 ± 0.34 | 6.12 ± 0.45 | 6.60 ± 0.92 | 6.09 ± 1.21 | - | - | **0.05** |
| Glycine | 56.09 ± 14.46[b] | 193.54 ± 41.24[a] | 201.88 ± 62.83[a] | 129.96 ± 75.90[ab] | - | - | **<0.01** |
| α-Aminobutyric acid | 6.24 ± 0.74[c] | 12.01 ± 1.66[ab] | 13.35 ± 2.72[a] | 8.03 ± 1.86[bc] | - | - | **<0.01** |
| Valine | 18.86 ± 4.69[c] | 134.91 ± 54.62[a] | 115.05 ± 45.29[ab] | 20.42 ± 4.64[bc] | - | - | **<0.01** |
| β-Aminoisobutyric acid | 6.29 ± 0.34 | 6.37 ± 0.44 | 6.90 ± 0.31 | 6.72 ± 0.70 | 0.65 | 0.34 | 0.44 |
| Leucine | 9.69 ± 1.44[b] | 71.83 ± 29.76[a] | 62.27 ± 25.54[a] | 9.93 ± 1.59[b] | - | - | **<0.01** |
| Isoleucine | 5.87 ± 0.80[b] | 49.57 ± 20.57[a] | 38.05 ± 16.03[a] | 6.08 ± 1.00[b] | - | - | **<0.01** |
| Threonine | 11.77 ± 1.85[b] | 33.76 ± 10.97[a] | 34.39 ± 11.16[a] | 12.72 ± 2.71[b] | - | - | **<0.01** |
| Serine | 16.00 ± 1.66[b] | 36.06 ± 8.30[a] | 31.13 ± 5.85[a] | 25.99 ± 11.12[b] | - | - | **<0.01** |
| Proline | 16.96 ± 3.72[b] | 42.09 ± 11.46[ab] | 48.00 ± 16.06[a] | 21.96 ± 4.28[ab] | - | - | **0.03** |
| Asparagine | 8.38 ± 0.42[bc] | 17.92 ± 4.01[a] | 15.02 ± 3.15[ab] | 9.93 ± 1.51[bc] | - | - | **<0.01** |
| Thiaproline | 1.23 ± 0.58 | 1.25 ± 0.59 | 1.24 ± 0.58 | 1.08 ± 0.52 | 0.07 | 0.57 | 0.54 |
| Aspartic acid | 7.49 ± 0.34 | 8.93 ± 0.83 | 8.45 ± 0.55 | 7.56 ± 0.47 | 0.54 | 0.59 | 0.22 |
| Methionine | 6.12 ± 0.29[b] | 10.94 ± 2.52[a] | 14.04 ± 4.81[a] | 6.20 ± 0.29[b] | - | - | **<0.01** |
| 4-Hydroxyproline | 19.36 ± 3.19 | 24.01 ± 4.12 | 33.85 ± 11.98 | 37.91 ± 14.47 | 0.74 | 0.37 | 0.39 |
| Glutamic acid | 35.15 ± 5.71 | 54.41 ± 9.78 | 45.77 ± 7.10 | 36.55 ± 6.17 | 0.11 | 0.41 | 0.10 |
| Phenylalanine | 10.95 ± 1.13[c] | 30.32 ± 8.02[a] | 30.73 ± 9.09[ab] | 12.32 ± 1.47[bc] | - | - | **<0.01** |
| α-Aminoadipic acid | 11.21 ± 0.46 | 11.82 ± 0.81 | 11.97 ± 0.73 | 13.86 ± 1.97 | 0.41 | 0.58 | 0.83 |
| Glutamine | 68.49 ± 11.29[b] | 171.93 ± 40.51[ab] | 240.97 ± 95.72[a] | 107.00 ± 34.60[ab] | - | - | **0.02** |
| Ornithine | 17.15 ± 1.15[b] | 37.30 ± 9.04[a] | 39.82 ± 10.76[a] | 20.34 ± 2.23[ab] | - | - | **0.01** |
| Glycine-proline | 2.77 ± 0.44[a] | 1.41 ± 0.44[b] | 1.69 ± 0.44[ab] | 2.08 ± 0.41[ab] | - | - | **0.05** |
| Lysine | 25.36 ± 2.72 | 52.33 ± 11.61 | 50.16 ± 11.41 | 28.32 ± 5.45 | - | - | **0.03** |
| Histidine | 15.97 ± 1.28 | 33.09 ± 8.85 | 34.22 ± 9.11 | 21.59 ± 6.90 | - | - | **0.03** |
| Hydroxylysine | 9.39 ± 0.24 | 9.47 ± 0.36 | 9.55 ± 0.31 | 10.15 ± 0.94 | 0.27 | 0.99 | 0.99 |
| Tyrosine | 11.63 ± 1.35[b] | 29.54 ± 7.70[a] | 28.63 ± 8.21[a] | 13.23 ± 1.65[ab] | - | - | **<0.01** |
| Proline-hydroxyproline | 47.74 ± 7.35 | 31.09 ± 7.35 | 42.15 ± 7.35 | 43.92 ± 6.86 | 0.31 | 0.62 | 0.21 |
| Tryptophan | 10.15 ± 0.36[b] | 22.71 ± 5.94[a] | 26.83 ± 9.89[ab] | 10.17 ± 0.38[b] | - | - | **<0.01** |
| Cystathionine | 0.18 ± 0.18 | 0.50 ± 0.34 | 0.89 ± 0.71 | 1.69 ± 1.58 | 0.91 | 0.56 | 0.43 |
| Cystine | 2.88 ± 0.76 | 5.34 ± 1.56 | 5.34 ± 1.53 | 2.38 ± 0.99 | 0.68 | 0.84 | 0.06 |

Data are presented as means ± standard error. Bolded *P* values are significant at $P \leq 0.05$. When there is a significant interaction, the *P* values for main effects of nutrition and treatment are not shown and replaced with a dash (-). Superscripts(a,b,c) denotes differences at $P \leq 0.05$. Concentrations are expressed in µmol/L. Experimental units: ADQ-CON, n = 6; RES-CON, n = 6; ADQ-MEL, n = 6; RES-MEL n = 7. Abbreviations: NUT, nutrition; TRT, treatment; NUT*TRT, nutrition by treatment interaction; ADQ-CON, adequately fed control; RES-CON, restricted fed control; ADQ-MEL, adequately fed melatonin supplemented; RES-MEL, restricted fed melatonin supplemented.

**Supplemental Table S5.** Individual amino acids in maternal plasma in the summer replicate.

| Amino Acid (μmol/L) | ADQ-CON | RES-CON | ADQ-MEL | RES-MEL | *P* value NUT | TRT | NUT*TRT |
|---|---|---|---|---|---|---|---|
| Alanine | 305.54 ± 67.55 | 319.15 ± 61.21 | 350.56 ± 73.22 | 271.85 ± 34.27 | 0.88 | 0.76 | 0.72 |
| Sarcosine | 7.70 ± 0.77 | 7.81 ± 0.37 | 8.16 ± 0.33 | 7.90 ± 0.87 | 0.48 | 0.86 | 0.39 |
| Glycine | 239.59 ± 15.61 | 285.05 ± 15.55 | 315.66 ± 43.59 | 268.75 ± 15.43 | 0.44 | 0.34 | 0.17 |
| α-Aminobutyric acid | 16.30 ± 2.91 | 22.50 ± 2.91 | 20.71 ± 2.91 | 19.81 ± 2.70 | 0.36 | 0.77 | 0.22 |
| Valine | 267.23 ± 15.70 | 202.20 ± 15.75 | 279.14 ± 16.37 | 247.25 ± 15.30 | **<0.01** | 0.08 | 0.32 |
| β-Aminoisobutyric acid | 8.00 ± 0.50 | 8.76 ± 0.80 | 9.88 ± 1.05 | 8.00 ± 0.50 | 0.50 | 0.42 | 0.16 |
| Leucine | 112.69 ± 12.47 | 95.02 ± 12.51 | 122.46 ± 12.64 | 131.99 ± 11.99 | 0.75 | 0.07 | 0.29 |
| Isoleucine | 73.77 ± 8.53 | 61.41 ± 8.56 | 76.23 ± 8.65 | 90.34 ± 8.20 | 0.92 | 0.08 | 0.14 |
| Threonine | 50.76 ± 3.86 | 43.17 ± 6.66 | 59.35 ± 9.66 | 49.72 ± 3.29 | 0.31 | 0.30 | 0.70 |
| Serine | 60.56 ± 5.94 | 55.33 ± 7.50 | 81.48 ± 25.53 | 54.02 ± 6.02 | 0.55 | 0.91 | 0.68 |
| Proline | 76.59 ± 11.20 | 82.43 ± 10.55 | 94.05 ± 17.95 | 75.33 ± 7.21 | 0.94 | 0.95 | 0.45 |
| Asparagine | 21.49 ± 2.07 | 23.19 ± 2.07 | 21.46 ± 2.35 | 26.01 ± 1.98 | 0.18 | 0.51 | 0.50 |
| Thiaproline | 1.00 ± 0.63 | 1.45 ± 0.65 | 1.95 ± 0.62 | 1.66 ± 0.59 | 0.39 | 0.50 | 0.18 |
| Aspartic acid | 12.07 ± 1.73 | 13.84 ± 2.41 | 29.00 ± 15.82 | 11.13 ± 1.10 | 0.97 | 0.55 | 0.36 |
| Methionine | 19.44 ± 1.81 | 19.89 ± 1.81 | 22.43 ± 1.81 | 21.76 ± 1.68 | 0.95 | 0.19 | 0.76 |
| 4-Hydroxyproline | 46.29 ± 14.08 | 41.55 ± 11.25 | 48.17 ± 14.62 | 27.97 ± 8.96 | 0.99 | 0.80 | 0.88 |
| Glutamic acid | 104.93 ± 25.62 | 132.44 ± 42.32 | 150.19 ± 41.91 | 85.26 ± 24.42 | 0.63 | 0.88 | 0.18 |
| Phenylalanine | 62.97 ± 8.37 | 62.16 ± 7.66 | 69.34 ± 10.71 | 51.96 ± 4.76 | 0.29 | 0.79 | 0.74 |
| α-Aminoadipic acid | 12.35 ± 1.46 | 11.15 ± 0.62 | 11.61 ± 0.57 | 10.58 ± 0.41 | 0.27 | 0.74 | 0.65 |
| Glutamine | 654.88 ± 182.88 | 702.49 ± 184.74 | 601.34 ± 131.57 | 536.72 ± 96.27 | 0.78 | 0.87 | 0.77 |
| Ornithine | 43.32 ± 3.02 | 50.55 ± 3.02 | 44.21 ± 3.72 | 56.51 ± 3.08 | **0.02** | 0.27 | 0.44 |
| Glycine-proline | 1.71 ± 0.82 | 1.94 ± 1.13 | 2.81 ± 1.34 | 0.46 ± 0.44 | 0.38 | 0.66 | 0.27 |
| Lysine | 60.13 ± 7.65 | 63.75 ± 7.63 | 76.54 ± 9.19 | 72.43 ± 7.77 | 0.98 | 0.12 | 0.63 |
| Histidine | 51.73 ± 6.20 | 52.77 ± 3.47 | 56.74 ± 7.06 | 60.43 ± 3.88 | 0.15 | 0.20 | 0.52 |
| Hydroxylysine | 9.05 ± 0.21[ab] | 9.34 ± 0.21[a] | 9.33 ± 0.26[ab] | 8.70 ± 0.22[b] | - | - | **0.05** |
| Tyrosine | 54.68 ± 4.83 | 53.68 ± 6.72 | 67.60 ± 10.22 | 52.24 ± 4.93 | 0.26 | 0.52 | 0.90 |
| Proline-hydroxyproline | 37.57 ± 9.38 | 38.95 ± 9.68 | 37.91 ± 9.35 | 27.49 ± 8.79 | 0.67 | 0.99 | 0.97 |
| Tryptophan | 40.13 ± 3.45 | 36.11 ± 3.45 | 41.21 ± 3.45 | 37.94 ± 3.21 | 0.30 | 0.68 | 0.91 |
| Cystathionine | 3.71 ± 1.91 | 7.16 ± 4.05 | 4.69 ± 1.86 | 2.96 ± 1.54 | 0.89 | 0.92 | 0.40 |
| Cystine | 22.03 ± 1.88 | 23.00 ± 1.88 | 25.34 ± 1.88 | 23.63 ± 1.75 | 0.84 | 0.30 | 0.48 |

Data are presented as means ± standard error. Bolded *P* values are significant at $P \leq 0.05$. When there is a significant interaction, the *P* values for main effects of nutrition and treatment are not shown and replaced with a dash (-). Superscripts(a,b,c) denotes differences at $P \leq 0.05$. Concentrations are expressed in μmol/L. Experimental units: ADQ-CON, n = 6; RES-CON, n = 6; ADQ-MEL, n = 6; RES-MEL n = 7. Abbreviations: NUT, nutrition; TRT, treatment; NUT*TRT, nutrition by treatment interaction; ADQ-CON, adequately fed control; RES-CON, restricted fed control; ADQ-MEL, adequately fed melatonin supplemented; RES-MEL, restricted fed melatonin supplemented.

**Supplemental Table S6.** Individual amino acids in fetal plasma in the summer replicate.

| Amino Acid (µmol/L) | ADQ-CON | RES-CON | ADQ-MEL | RES-MEL | *P* value NUT | TRT | NUT*TRT |
|---|---|---|---|---|---|---|---|
| Alanine | 450.59 ± 37.25 | 440.04 ± 37.26 | 544.15 ± 45.38 | 462.58 ± 35.79 | 0.27 | 0.14 | 0.40 |
| Sarcosine | 9.91 ± 0.52[ab] | 8.69 ± 0.52[b] | 9.23 ± 0.52[ab] | 10.25 ± 0.48[a] | - | - | **0.04** |
| Glycine | 276.83 ± 16.80 | 256.63 ± 36.04 | 289.56 ± 14.23 | 323.04 ± 22.51 | 0.26 | 0.16 | 0.35 |
| α-Aminobutyric acid | 21.76 ± 2.47 | 27.09 ± 2.48 | 27.77 ± 2.58 | 33.94 ± 2.41 | **0.03** | **0.02** | 0.87 |
| Valine | 251.03 ± 17.16 | 183.96 ± 32.23 | 272.30 ± 21.71 | 224.73 ± 11.98 | **<0.01** | 0.27 | 0.84 |
| β-Aminoisobutyric acid | 9.17 ± 0.53 | 9.69 ± 0.53 | 9.59 ± 0.54 | 11.47 ± 0.50 | **0.03** | **0.05** | 0.23 |
| Leucine | 90.61 ± 6.23 | 70.77 ± 12.63 | 92.54 ± 3.02 | 85.53 ± 2.21 | 0.10 | 0.54 | 0.79 |
| Isoleucine | 56.31 ± 4.22 | 42.17 ± 7.83 | 59.75 ± 3.02 | 57.00 ± 2.00 | 0.06 | 0.09 | 0.43 |
| Threonine | 47.46 ± 5.95 | 45.23 ± 12.02 | 48.70 ± 4.43 | 36.77 ± 2.92 | **<0.01** | 0.86 | 0.09 |
| Serine | 71.43 ± 5.28 | 59.20 8.21 | 70.93 ± 3.05 | 75.72 ± 5.22 | 0.76 | 0.22 | 0.24 |
| Proline | 105.04 ± 6.25 | 99.15 ± 15.28 | 105.15 ± 8.97 | 114.13 ± 4.29 | 0.48 | 0.58 | 0.48 |
| Asparagine | 24.88 ± 1.19 | 22.80 ± 2.85 | 23.86 ± 1.30 | 25.75 ± 0.72 | - | - | **0.05** |
| Thiaproline | 0.96 ± 0.61 | 1.95 ± 0.62 | 0.97 ± 0.61 | 1.26 ± 0.59 | 0.66 | 0.22 | 0.40 |
| Aspartic acid | 13.51 ± 1.58 | 14.47 ± 1.59 | 15.23 ± 1.61 | 19.64 ± 1.51 | 0.10 | **0.04** | 0.31 |
| Methionine | 20.49 ± 2.21 | 20.00 ± 2.21 | 23.13 ± 2.21 | 19.52 ± 2.05 | 0.36 | 0.62 | 0.48 |
| 4-Hydroxyproline | 72.81 ± 5.86 | 73.41 ± 5.88 | 79.11 ± 5.94 | 84.03 ± 5.63 | 0.64 | 0.16 | 0.72 |
| Glutamic acid | 139.15 ± 24.30 | 139.69 ± 24.30 | 191.46 ± 24.30 | 206.71 ± 22.29 | 0.74 | **0.02** | 0.77 |
| Phenylalanine | 81.82 ± 4.24 | 72.90 ± 12.68 | 83.59 ± 5.47 | 69.99 ± 4.59 | 0.21 | 0.33 | 0.41 |
| α-Aminoadipic acid | 12.03 ± 0.33 | 11.56 ± 0.38 | 12.89 ± 0.79 | 11.59 ± 0.29 | 0.22 | 0.88 | 0.90 |
| Glutamine | 900.51 ± 99.07 | 858.60 ± 99.35 | 796.93 ± 103.26 | 1140.78 ± 96.52 | 0.15 | 0.37 | 0.08 |
| Ornithine | 51.02 ± 4.84 | 52.27 ± 4.84 | 53.73 ± 5.00 | 59.61 ± 4.58 | 0.47 | 0.30 | 0.64 |
| Glycine-proline | 2.52 ± 0.56 | 3.14 ± 0.50 | 4.08 ± 1.26 | 4.21 ± 0.72 | 0.27 | 0.14 | 0.85 |
| Lysine | 76.16 ± 7.56 | 62.56 ± 7.56 | 75.75 ± 8.88 | 71.16 ± 7.31 | 0.28 | 0.60 | 0.58 |
| Histidine | 61.94 ± 4.6[a] | 53.04 ± 4.63[ab] | 43.92 ± 4.8[b] | 62.18 ± 4.50[a] | - | - | **<0.01** |
| Hydroxylysine | 9.84 ± 0.15 | 9.64 ± 0.15 | 9.83 ± 0.16 | 9.83 ± 0.14 | 0.52 | 0.54 | 0.54 |
| Tyrosine | 66.99 ± 7.69 | 62.38 ± 7.69 | 80.53 ± 7.69 | 59.99 ± 7.14 | 0.11 | 0.47 | 0.30 |
| Proline-hydroxyproline | 52.58 ± 4.45 | 61.13 ± 4.46 | 51.82 ± 4.64 | 64.26 ± 4.34 | **0.03** | 0.79 | 0.68 |
| Tryptophan | 45.18 ± 3.70 | 40.22 ± 3.71 | 42.87 ± 3.86 | 49.74 ± 3.60 | 0.80 | 0.34 | 0.14 |
| Cystathionine | 6.20 ± 1.19 | 9.04 ± 1.93 | 8.04 ± 2.51 | 10.35 ± 2.00 | 0.10 | 0.46 | 0.38 |
| Cystine | 20.14 ± 0.99 | 21.39 ± 2.75 | 23.58 ± 1.85 | 21.72 ± 0.80 | 0.80 | **0.04** | 0.95 |

Data are presented as means ± standard error. Bolded *P* values are significant at $P \leq 0.05$. When there is a significant interaction, the *P* values for main effects of nutrition and treatment are not shown and replaced with a dash (-). Superscripts(a,b,c) denotes differences at $P \leq 0.05$. Concentrations are expressed in µmol/L. Experimental units: ADQ-CON, n = 6; RES-CON, n = 6; ADQ-MEL, n = 6; RES-MEL n = 7. Abbreviations: NUT, nutrition; TRT, treatment; NUT*TRT, nutrition by treatment interaction; ADQ-CON, adequately fed control; RES-CON, restricted fed control; ADQ-MEL, adequately fed melatonin supplemented; RES-MEL, restricted fed melatonin supplemented.

**Supplemental Table S7.** Individual amino acids in amnion in the summer replicate.

| Amino Acid (µmol/L) | ADQ-CON | RES-CON | ADQ-MEL | RES-MEL | NUT | TRT | NUT*TRT |
|---|---|---|---|---|---|---|---|
| Alanine | 247.61 ± 120.46 | 161.39 ± 22.29 | 149.37 ± 31.52 | 139.67 ± 36.38 | 0.70 | 0.45 | 0.70 |
| Sarcosine | 7.68 ± 0.96 | 6.34 ± 0.46 | 6.30 ± 0.63 | 8.53 ± 2.67 | 0.30 | 0.42 | 0.90 |
| Glycine | 190.46 ± 43.19 | 201.58 ± 48.70 | 189.75 ± 43.29 | 183.55 ± 74.41 | 0.47 | 0.38 | 0.42 |
| α-Aminobutyric acid | 13.57 ± 4.19 | 13.54 ± 2.19 | 9.19 ± 1.07 | 9.58 ± 1.52 | 0.88 | 0.06 | 0.25 |
| Valine | 196.32 ± 69.56 | 177.73 ± 52.55 | 107.88 ± 37.62 | 101.15 ± 49.88 | 0.77 | 0.13 | 0.95 |
| β-Aminoisobutyric acid | 7.74 ± 0.59 | 6.88 ± 0.35 | 6.83 ± 0.46 | 8.94 ± 2.26 | 0.35 | 0.39 | 0.87 |
| Leucine | 81.12 ± 29.19 | 88.11 ± 25.84 | 53.34 ± 18.43 | 49.84 ± 24.55 | 0.97 | 0.08 | 0.74 |
| Isoleucine | 53.77 ± 20.56 | 58.27 ± 17.52 | 34.97 ± 13.28 | 33.91 ± 17.42 | 0.79 | 0.09 | 0.78 |
| Threonine | 37.60 ± 10.35 | 38.27 ± 9.12 | 31.28 ± 7.30 | 25.38 ± 7.86 | 0.61 | 0.17 | 0.64 |
| Serine | 44.72 ± 8.86 | 36.26 ± 7.48 | 31.96 ± 8.06 | 31.41 ± 7.48 | 0.58 | 0.28 | 0.63 |
| Proline | 61.86 ± 21.02 | 47.55 ± 8.76 | 33.03 ± 8.18 | 40.54 ± 11.06 | 0.93 | 0.23 | 0.54 |
| Asparagine | 18.63 ± 3.49 | 16.77 ± 2.78 | 14.06 ± 1.99 | 13.53 ± 2.48 | 0.54 | 0.24 | 0.97 |
| Thiaproline | 2.35 ± 0.59 | 0.43 ± 0.43 | 0.96 ± 0.61 | 0.82 ± 0.53 | **0.04** | 0.19 | 0.15 |
| Aspartic acid | 10.17 ± 1.00 | 7.93 ± 0.39 | 8.41 ± 0.62 | 8.17 ± 0.47 | 0.14 | 0.32 | 0.16 |
| Methionine | 14.68 ± 3.04 | 12.86 ± 2.34 | 10.88 ± 1.83 | 10.27 ± 2.39 | 0.52 | 0.15 | 0.83 |
| 4-Hydroxyproline | 31.06 ± 12.39 | 22.75 ± 4.85 | 20.70 ± 4.88 | 60.85 ± 42.63 | 0.46 | 0.77 | 0.89 |
| Glutamic acid | 65.25 ± 14.49 | 41.69 ± 5.71 | 38.01 ± 4.80 | 36.84 ± 5.12 | **0.01** | 0.17 | 0.91 |
| Phenylalanine | 41.51 ± 13.60 | 36.18 ± 7.14 | 24.79 ± 5.54 | 22.09 ± 7.05 | 0.71 | 0.11 | 0.94 |
| α-Aminoadipic acid | 13.88 ± 1.34 | 10.55 ± 0.41 | 12.93 ± 2.62 | 12.97 ± 2.74 | 0.11 | 0.52 | 0.40 |
| Glutamine | 232.72 ± 79.70 | 208.12 ± 34.34 | 173.71 ± 38.50 | 175.49 ± 46.20 | 0.82 | 0.38 | 0.60 |
| Ornithine | 38.05 ± 8.07 | 36.60 ± 6.47 | 26.64 ± 3.50 | 35.79 ± 10.44 | 0.88 | 0.19 | 0.91 |
| Glycine-proline | 1.34 ± 0.66 | 1.49 ± 0.67 | 2.09 ± 0.95 | 1.93 ± 0.59 | 0.67 | 0.31 | 0.23 |
| Lysine | 50.89 ± 6.56 | 49.13 ± 8.47 | 42.48 ± 8.06 | 57.68 ± 27.69 | 0.43 | 0.15 | 0.58 |
| Histidine | 41.62 ± 8.00 | 38.57 ± 6.12 | 34.90 ± 6.74 | 27.97 ± 5.62 | 0.28 | 0.12 | 0.59 |
| Hydroxylysine | 10.38 ± 1.18 | 8.89 ± 0.10 | 9.74 ± 0.79 | 12.46 ± 3.52 | 0.44 | 0.92 | 0.75 |
| Tyrosine | 41.27 ± 10.79 | 34.34 ± 6.68 | 24.38 ± 4.60 | 21.81 ± 6.58 | 0.31 | **0.02** | 0.42 |
| Proline-hydroxyproline | 46.93 ± 15.60 | 31.94 ± 7.82 | 44.52 ± 4.60 | 43.37 ± 10.37 | 0.66 | 0.41 | 0.06 |
| Tryptophan | 28.86 ± 7.86 | 25.54 ± 5.26 | 19.89 ± 4.22 | 18.55 ± 5.56 | 0.19 | 0.07 | 0.79 |
| Cystathionine | 2.17 ± 1.98 | 0.30 ± 0.19 | 0.89 ± 0.71 | 6.26 ± 6.26 | 0.64 | 0.54 | 0.73 |
| Cystine | 8.15 ± 1.31 | 11.40 ± 2.55 | 8.94 ± 1.79 | 7.85 ± 1.87 | 0.83 | 0.54 | 0.29 |

Data are presented as means ± standard error. Bolded *P* values are significant at $P \leq 0.05$. When there is a significant interaction, the *P* values for main effects of nutrition and treatment are not shown and replaced with a dash (-). Superscripts(a,b,c) denotes differences at $P \leq 0.05$. Concentrations are expressed in µmol/L. Experimental units: ADQ-CON, n = 6; RES-CON, n = 6; ADQ-MEL, n = 6; RES-MEL n = 7. Abbreviations: NUT, nutrition; TRT, treatment; NUT*TRT, nutrition by treatment interaction; ADQ-CON, adequately fed control; RES-CON, restricted fed control; ADQ-MEL, adequately fed melatonin supplemented; RES-MEL, restricted fed melatonin supplemented.
